# Global Analysis of mRNA Half-Lives and *de novo* Transcription in a Dinoflagellate, *Karenia brevis*

**DOI:** 10.1371/journal.pone.0066347

**Published:** 2013-06-11

**Authors:** Jeanine S. Morey, Frances M. Van Dolah

**Affiliations:** Marine Biotoxins Program, National Oceanic and Atmospheric Administration, National Ocean Service, Center for Coastal Environmental Health and Biomolecular Research, Charleston, South Carolina, United States of America; University of Melbourne, Australia

## Abstract

Dinoflagellates possess many physiological processes that appear to be under post-transcriptional control. However, the extent to which their genes are regulated post-transcriptionally remains unresolved. To gain insight into the roles of differential mRNA stability and *de novo* transcription in dinoflagellates, we biosynthetically labeled RNA with 4-thiouracil to isolate newly transcribed and pre-existing RNA pools in *Karenia brevis*. These isolated fractions were then used for analysis of global mRNA stability and *de novo* transcription by hybridization to a *K. brevis* microarray. Global *K. brevis* mRNA half-lives were calculated from the ratio of newly transcribed to pre-existing RNA for 7086 array features using the online software HALO (Half-life Organizer). Overall, mRNA half-lives were substantially longer than reported in other organisms studied at the global level, ranging from 42 minutes to greater than 144 h, with a median of 33 hours. Consistent with well-documented trends observed in other organisms, housekeeping processes, including energy metabolism and transport, were significantly enriched in the most highly stable messages. Shorter-lived transcripts included a higher proportion of transcriptional regulation, stress response, and other response/regulatory processes. One such family of proteins involved in post-transcriptional regulation in chloroplasts and mitochondria, the pentatricopeptide repeat (PPR) proteins, had dramatically shorter half-lives when compared to the arrayed transcriptome. As transcript abundances for PPR proteins were previously observed to rapidly increase in response to nutrient addition, we queried the newly synthesized RNA pools at 1 and 4 h following nitrate addition to N-depleted cultures. Transcriptome-wide there was little evidence of increases in the rate of *de novo* transcription during the first 4 h, relative to that in N-depleted cells, and no evidence for increased PPR protein transcription. These results lend support to the growing consensus of post-transcriptional control of gene expression in dinoflagellates.

## Introduction

Phytoplankton are essential primary producers, responsible for up to 70% of the world's oxygen production. Dinoflagellates are a major component of both marine and freshwater phytoplankton and, as approximately half are photosynthetic, they are key contributors to the base of aquatic food webs. However, many dinoflagellates also produce potent toxins and are responsible for harmful algal blooms (HABs). As HABs have significant ecological, human health, and economic impacts [1] much recent attention has been focused on their molecular biology to better understand the mechanisms underlying bloom dynamics and toxicity for improved coastal management and forecasting. These unicellular protists have several unusual nuclear traits, including exceptionally large genomes of 3–245×10^6^ kbp [2]. Dinoflagellate genes are often present in tandem arrays and lack recognizable transcription regulatory elements [3]. Tandemly arrayed gene copies appear to be transcribed into polycistronic mRNAs [3], although this has recently been challenged [4] and remains open to debate. All nuclear encoded mRNAs examined possess an identical 5′ trans-spliced spliced leader (SL) sequence [3], [5], [6]. SL *trans*-splicing was first described in the kinetoplastid, *Trypanosoma brucei* [7]. In trypanosomes, messages are transcribed as polycistronic pre-mRNAs that are processed to mature monocistronic messages by the trans-splicing mechanism. Nearly all genes are constitutively transcribed, relying on post-transcriptional control for their regulation [8]. Dinoflagellates also appear to have reduced levels of transcriptional control relative to other eukaryotes (reviewed in [9]). However, the extent to which post-transcriptional control of gene expression occurs in dinoflagellates remains uncertain. Circadian controlled bioluminescence rhythms in *Lingulodinium polyedrum* do not depend upon changes in mRNA levels of luciferin binding protein or luciferase [10], [11] nor do circadian changes in GAPDH [12], peridinin-chlorophyl a-binding protein [13], or superoxide dismutase [14] protein levels. Likewise, in *Karenia brevis* cell cycle genes, typically under transcriptional control in most organisms, appear to be post-transcriptionally regulated [15], [16]. Microarray studies report little evidence of change in transcript abundance of genes associated with acute stress responses [17] or responsive to nitrogen or phosphorus limitation [18]. Using massively parallel signature sequencing (MPSS), Moustafa et al. [19] found 73% of the transcriptome of *Alexandrium tamarense* unchanged under a variety of conditions. A similar magnitude of transcriptome restructuring is found concurrent with the entry into stationary phase in *K. brevis* (29%) [20] and *Alexandrium minutum* (36%) [21]. However, microarray and MPSS cannot determine what mechanism(s) are responsible for the measured changes in transcript abundance.

The steady-state abundance of mRNAs in a cell is determined by their relative rates of transcription and degradation [22], [23]. Thus changes in transcript abundance measured by microarray, high throughput transcriptome sequencing, or qPCR in the studies cited above may reflect perturbations to either of these mechanisms. Surveys of RNA stability in a number of eukaryotes reveal a wide range of RNA half-lives that vary over at least two orders of magnitude [23]. In general RNA half-lives are related to their physiological roles [24], wherein housekeeping genes typically have long RNA half-lives while proteins needed for short durations often have messages with short half-lives. Highly represented among the genes with rapid mRNA turnover are those known to be transcriptionally inducible [25]. Therefore, to gain a better understanding of the processes underlying the regulation of the dinoflagellate transcriptome, this study examines global message stability and *de novo* transcription in *K. brevis* utilizing biosynthetic labeling of newly transcribed RNA.

RNA stability is often determined using transcription inhibitors to block *de novo* transcription and then monitoring the decay of specific messages over time. A disadvantage of this approach is that transcription inhibition has been shown to artificially stabilize many messages, leading to over estimation of RNA half-lives [24], [25], [26]. Pulse-chase experiments can also be used to measure decay rates of individual RNAs following radiolabeling of RNA, but tend to be imprecise for medium- to long-lived messages [27]. An alternative approach is to biosynthetically label newly synthesized RNA with 4-thiouracil or 4-thiouridine, which are readily incorporated and do not impact message stability (reviewed in [28]). Thiol-specific biotinylation, followed by streptavidin-coated magnetic bead separation, enables total RNA to be separated into pools of pre-existing and newly synthesized (thiolated) RNA that are amenable to transcriptome-level downstream applications such as microarrays or RNA-seq [22], [24], [28], [29], [30], [31], [32], [33], [34], [35]. In addition to providing a means to identify transcriptionally activated genes, global RNA half-lives can be determined, capitalizing on the fact that newly synthesized RNA and pre-existing RNA pools sum to the total RNA pool. Under steady state conditions RNA synthesis compensates for RNA decay, consequently *de novo* synthesis rates must be much higher for short-lived transcripts than for stable transcripts. Therefore, RNA half-lives can be calculated from the ratios of either newly synthesized/total RNA, pre-existing RNA/total RNA, or newly synthesized/pre-existing RNA, and the duration of labeling [22], [24], [28], [31]. This approach results in precise and reproducible data independent of the individual mRNA half-lives [22].

In this study we applied biosynthetic nucleotide labeling to characterize global mRNA stability in *K. brevis* and to examine the role of *de novo* transcription in achieving short-term changes in transcript abundances by microarray analysis. Overall, *K. brevis* RNA half-lives were unusually stable, with a median half-life of 33 h, although as in most organisms studied, half-lives ranged over two orders of magnitude, from 42 minutes to greater than 144 h. Consistent with measurements from other organisms, there is a predominance among highly stable messages of genes that are involved in core cellular processes, including energy metabolism and transport. Shorter-lived transcripts include many genes involved in transcriptional regulation, stress response, and other response/regulatory processes. Microarray analysis of newly synthesized RNA following nitrate addition to N-depleted cultures revealed little change in the rate of *de novo* transcription of genes found previously to increase in abundance within 4 h of nitrate addition [18]. These results provide further support for post-transcriptional mechanisms such as differential RNA stability driving changes in gene expression in dinoflagellates.

## Materials and Methods

### *K. brevis* Culture Conditions

Batch cultures of *K. brevis* (Wilson isolate) were maintained in 1 L bottles in *f*/2 medium using 20 µm filtered, autoclaved natural seawater (salinity 36) with the following modifications: ferric sequestrene was substituted for EDTA·Na_2_ and FeCl_3_·6H_2_O and 0.01 mM Na_2_SiO_3_ was added. Nitrogen-limited cultures were adapted to 10 µM nitrate, approximately 1.1% of the nitrate concentration in nutrient replete cultures, by a minimum of six serial log phase transfers prior to experimental treatments. N-depleted cultures were achieved by allowing these cultures to enter stationary phase. Illumination from cool white lights was supplied at approximately 175–215 µmol photons·m^−2^·s^−1^ on a 16∶8 h light∶dark cycle at 25±1°C. All experiments were initiated and cultures harvested during the light phase of the diel cycle.

### Biosynthetic Labeling and Extraction of RNA

To determine if *K. brevis* is amenable to biosynthetic labeling of newly synthesized RNA by 4-thiouracil (4tU, Sigma, St. Louis, MO) or 4-thiouridine (4sU, LKT Laboratories, Inc., St. Paul, MN), cultures were exposed to 0.02, 0.05, or 0.2 mM 4tU (in DMSO) or 0.2, 1, or 2 mM 4sU (in water) for 5 m to 20 h. To determine the half-lives of *K. brevis* mRNAs, one liter log-phase cultures (n = 6) were exposed to 0.2 mM 4tU for 2 h. To determine the transcriptional response to increased nitrate concentrations, sodium nitrate was used to add 155 µM NO_3_ to 6 N-depleted 1 L cultures, as in Morey et al. [18]. 4-thiouracil (0.2 mM) was then added to triplicate cultures for 1 h from 0 h–1 h post N-addition and to triplicate cultures from 3 h–4 h post N-addition. Newly transcribed messages were labeled in parallel by the incorporation of 0.2 mM 4tU for 1 h in triplicate N-depleted cultures. Cultures were harvested by centrifugation at 600× *g* for 10 m and total RNA was extracted using Tri-Reagent (Molecular Research Center, Inc., Cincinnati, OH) according to the manufacturer's protocol with ethanol precipitation. RNA was resuspended in nuclease-free water, treated with Promega's (Madison, WI) RQ1 RNase-free DNase, and further purified using an RNeasy mini column according to manufacturer's protocol (Qiagen, Valencia, CA). RNA was then quantified using a NanoDrop ND-1000 (Wilmington, DE) and qualified on an Agilent 2100 Bioanalyzer (Santa Clara, CA).

### Biotinylation, Detection, and Fractionation of RNA

Total RNA was biotinylated according to the methods of Dölken et al. [22] with minor modifications, using Pierce EZ-Link Biotin-HPDP (Rockford, IL) for 3 h at 60°C in the dark with rotation. Two microliters of 1 mg·mL^−1^ Biotin-HPDP in DMF were added to the labeling reaction per 1 µg of RNA. The labeling was carried out in 10 mM Tris (pH 7.4) and 1 mM EDTA in a final volume 5X that of the Biotin-HPDP in the reaction. RNA was then precipitated by the addition of 1/10 the reaction volume of 5 M NaCl and 1.1 volumes of isopropanol and centrifugation at 20,000× *g* for 20 m at room temperature. The pellet was washed in 75% EtOH, centrifuged at 20,000× *g* for 5 m at room temperature, resuspended in nuclease-free water, and quantified and qualified as described previously.

To determine how quickly 4tU is incorporated into *K. brevis* RNA, total RNA was biotinylated, heat denatured, centrifuged at 20,000× *g* for 1 m to remove free biotin, and bound to positively charged nylon membrane (Roche, Indianapolis, IN) with alkaline binding buffer (10 mM NaOH, 1 mM EDTA) using a slot blot apparatus [22]. The membrane was blocked with PBS, pH 7.5, 10% SDS, 1 mM EDTA for 30 m at room temperature and probed with Pierce High Sensitivity Streptavidin – HRP at 1∶1000 in blocking buffer for 15 m at room temperature. The blot was washed 2×10 m each in PBS, pH 7.5 containing decreasing amounts of SDS (10%, 1%, 0.1%) and detected using Pierce SuperSignal West Pico Chemiluminescent Substrate.

Following biotinylation, total RNA was fractionated into two pools, newly synthesized thiolated RNA and pre-existing non-thiolated RNA using Dynabeads MyOne Streptavidin C1 magnetic beads (Life Technologies, Grand Island, NY). One microliter of beads was used per 160 ng of RNA and manufacturer's protocols were followed for immobilization of nucleic acids with the addition of 0.1% Tween 20 to the binding and washing buffer (BWT). Following immobilization, the supernatant containing unlabeled RNA was saved and, following isopropanol precipitation, constituted the pre-existing RNA fraction. The beads were then washed three times in 3X the original volume of beads with 1X BWT at 65°C, followed by 3 washes at room temperature and a final wash with 1∶10 1X BWT at room temp. Biotinylated RNA was released from the beads by adding 1X volume of 5% β-mercaptoethanol and incubating 5 m at room temperature with rotation. The supernatant was saved and another 1X volume of 5% β-mercaptoethanol was added to the beads and incubated at 60°C for 10 m with occasional mixing. This supernatant was added to the previous elution and, following isopropanol precipitation, constituted the newly synthesized RNA fraction.

### Microarray Analysis

A *K. brevis* oligonucleotide microarray containing 10,263 unique 60-mer gene probes [36], [37] was used for these studies, employing a one-color protocol. For half-life studies total, newly synthesized, and pre-existing RNA pools were labeled. For the nitrate addition study only the newly synthesized RNA was labeled. Based on bioanalyzer profiles, total and pre-existing RNA pools were treated as total RNA samples, whereas newly synthesized RNA pools were treated as mRNA samples, as described in Dölken et al. [22]. Agilent's Low Input Quick Amp Labeling Kit was used to amplify and label total RNA (100 ng) or mRNA (25 ng) with Cy3 dye. The amplified labeled RNA was quantified using a NanoDrop ND-1000 and 480 ng of Cy3 labeled targets were hybridized to the array for 17 h at 60°C. After hybridization, arrays were washed according to the manufacturer's protocol and imaged using an Agilent microarray scanner. Images were extracted with Agilent Feature Extraction version 9.5.3.1 using a rank consistency filter and a combination linear and LOWESS normalization algorithm.

Half-Life Calculation: Normalized data for each biological replicate of total, pre-existing, or newly synthesized RNA were uploaded to the freely available software HALO (Half-life Organizer, www.bio.ifi.lmu.de/software/HALO) [31]. Data were filtered, where a Feature Extraction p≤0.0001 was required in all data sets (n = 18) for inclusion of a probe in further analyses to assure only high quality data was utilized. Since newly transcribed and pre-existing RNA should sum up to total RNA, a negative linear correlation should exist between the newly transcribed/total and pre-existing/total RNA ratios. Data was therefore normalized by linear regression in HALO. This normalization step is necessary because the amount of template RNA differs between the three different RNA pools, therefore standard microarray normalization methods which assume equal overall intensities are insufficient [31]. A probe quality score (PQS) assessing the distance of each feature from the regression line was then calculated for each feature, Features with PQSs greater than 1 were filtered out and normalization, PQS calculation, and data filtering were repeated [31]. Half-lives were then calculated for each regression-normalized feature using newly synthesized/pre-existing RNA as well as newly synthesized/total RNA and pre-existing/total RNA. The minimum half-life was set at 1 m and the maximum established empirically at 8640 m, a time at which less than 1.5% of features exceeded the maximum.

Transcriptional Response to Nitrate: Following feature extraction, microarray data were analyzed with Rosetta Resolver version 7.2 gene expression analysis system (Rosetta Biosoftware, Cambridge, MA). Based on the Rosetta error model designed for the Agilent platform, a composite array was generated for newly-synthesized RNA at each time point following N-addition (1 and 4 h) from triplicate arrays (representing three biological replicates), in which the data for each feature underwent a normalization, intensity averaging, and error estimation based on data from the replicate arrays making up the composite [38]. The composite arrays were then used to build ratios at each time point, relative to newly transcribed RNA over a 1 h period in the N-depleted cultures.

Blast2GO Annotation: Blast2GO [39], [40] was used to partially automate annotation, assign gene ontology (GO) terms, and test for enrichment. Additional annotation was carried out using the Yeast GO slim application. Fisher's exact enrichment tests were carried out on GO terms for annotated features relative to all features on the microarray in Blast2GO.

### RNA Stability Following Transcription Inhibition

Tube cultures (25 mL) of *K. brevis* were grown to mid-log phase under standard culture conditions (described above). Triplicate tubes were then treated with either 2 µg·mL^−1^ actinomycin D or DMSO carrier for 12, 24 or 48 h. Total RNA was harvested at each time point as described above, quantified using a NanoDrop ND-1000 (Wilmington, DE) and duplicate reverse transcription reactions were carried out using 100 ng total RNA with an oligo(dT) primer using Ambion's RETROscript Kit (Austin, TX). Primer pairs specific for the contig of interest were designed (Table S1) for qPCR on an ABI 7500 using the ABI Power SYBR Green master mix (Applied Biosystems, Foster City, CA). The optimal annealing temperature for each primer set was determined prior to the analysis of experimental samples. The efficiency of each primer set was determined using a standard curve of cDNA from *K. brevis*. The specificity of each primer set and size of the amplicon were verified by analysis with an Agilent Bioanalyzer 2100 and further confirmed by melt curve analysis. A cycle threshold (C_t_) was assigned at the beginning of the logarithmic phase of PCR amplification and the difference in the C_t_ values of the actinomycin D treated and carrier control samples were used to determine the relative expression of the gene in each time point.

## Results and Discussion

### *K. brevis* readily incorporates 4-thiouracil into RNA

**Figure 1:**
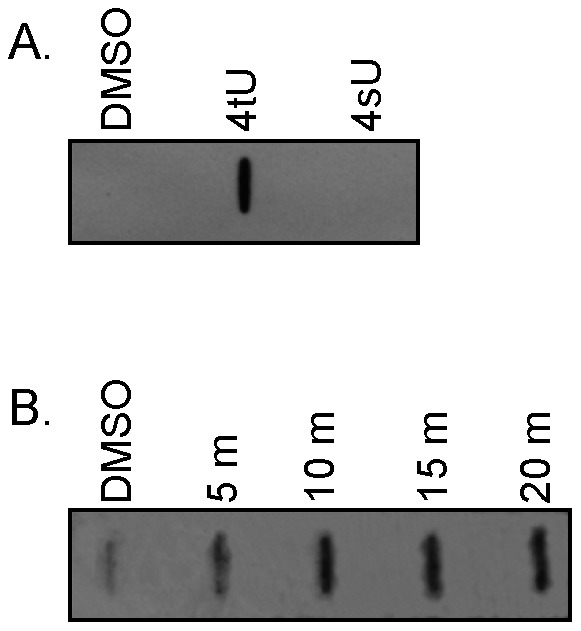
Incorporation of 4sU and 4tU into *K.* *brevis* RNA. **A.** Slot blots of biotinylated total RNA (1 µg) show that *K. brevis* specifically incorporates 0.2 mM 4-thiouracil (4tU), but not 0.2 mM 4-thiouridine (4sU), during a 20 h exposure. **B.** Following exposure to 0.2 mM 4tU, incorporation into RNA is detected in as little as 5 minutes (10 µg RNA loaded per slot). A representative blot is shown from multiple studies. An equal amount total RNA from cells treated with DMSO (carrier), processed similarly, served as a negative control to measure non-specific binding of biotin. Detection was with streptavidin-HRP.

Methods for labeling and purifying newly synthesized RNA amenable to high-throughput downstream analyses have been employed in a variety of eukaryotes using either 4-thiouracil (4tU) or 4-thiouridine (4sU) [22], [24], [28], [29], [30], [31], [32], [33], [34], [35]. Incorporation of 4tU requires the activity of uracil phosphoribosyltransferase (UPRT), a key enzyme in the pyrimidine salvage pathway for recycling uracil to uridine monophosphate (UMP). In contrast, 4sU is incorporated by uridine kinase (UK), which recycles uridine by phosphorylation to form UMP. Both UK and UPRT are widely found in prokaryotes and eukaryotes. However, in many eukaryotes, UK has higher activity than UPRT leading to more efficient salvage of uridine rather than uracil [41]. To date, only *Toxoplasma gondii* and yeast have demonstrated the ability to utilize 4tU as a substrate for RNA synthesis [28], [29], [30]. Because we had no *a priori* knowledge of the pyrimidine salvage pathway in the dinoflagellate, we first assessed the ability *of K. brevis* to incorporate 4tU and/or 4sU into newly synthesized RNA. Cultures of *K. brevis* were exposed to increasing concentrations of 4tU (0.02, 0.05, or 0.2 mM) or 4sU (0.2, 1.0, or 2.0 mM) over a range of times (5 m to 20 h). These concentrations have previously been shown to yield measurable incorporation into RNA by *T. gondii* (4tU) [30] and a variety of eukaryotic cell types (4sU) [22]. Following thiol-specific biotinylation, the incorporation of thiolated nucleotides into mRNA was measured by transferring total RNA to nylon membranes using a slot blot and detecting biotinylated RNA with streptavidin-HRP. We observed incorporation of 0.2 mM 4tU, but not 4sU (Figure 1a). Incorporation of a 10-fold higher concentration of 4sU was still undetectable, even after 20 h (data not shown). Measurable 4tU was incorporated into *K. brevis* mRNA in as little as 5 minutes (Figure 1b). The *K. brevis* transcriptome contains a putative UPRT gene (KbUPRT1, Genebank Accession No. KC862324) with highest homology to UPRT in *Aureococcus* (BLASTx e-value  = 1E-67), as well as a gene encoding a dual domain UK-UPRT protein (KbUK-UPRT1, Genebank Accession No. KC862325) with highest homology to *Arabidopsis thaliana* (BLASTx e-value  = 1E-174). UK-UPRT fusion genes encoding bifunctional proteins with both UK and UPRT activities are found across eukaryotic the tree of life, but are notably absent from the nearest neighbors to dinoflagellates, the apicomplexans, except for *Cryptosporidium parvum*, and have not previously been identified in dinoflagellates [42]. Based on the incorporation of 4tU and the presence of UPRT genes in the *K. brevis* transcriptome, it appears that *K. brevis* contains a pyrimidine salvage pathway that utilizes UPRT activity.

**Figure 2:**
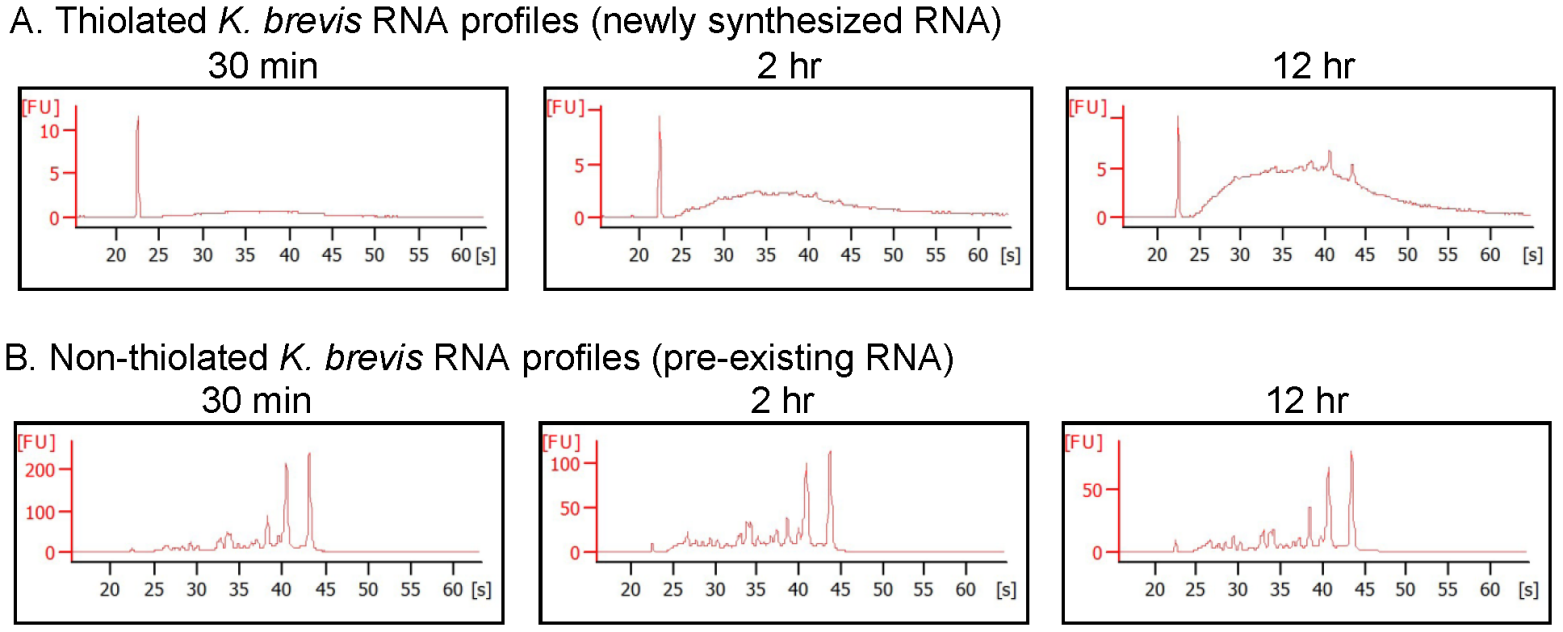
Representative bioanalyzer profiles of pre-existing and newly synthesized RNA fractions. **A**. Following 4-thiouracil (4tU) incorporation, biotinylation, and streptavidin-magnetic bead purification, newly synthesized RNA fractions are comparable to purified mRNA samples from *K. brevis*. With increasing labeling time, synthesis of ribosomal RNA becomes discernible, with peaks at approximately 41 and 44 s. The peak at 23 s is the manufacturer supplied internal marker (visually absent in B because of differences in the Y-axis scale). **B**. Bioanalyzer profiles of pre-existing RNA are similar to typical *K. brevis* total RNA profiles with dominant ribosomal peaks at approximately 41 and 44 s.

When the newly synthesized RNA was separated from pre-existing RNA by thiol-specific biotinylation, we observed expected bioanalyzer profiles (Figure 2a), wherein early time points contained only mRNAs, visible as a broad peak increasing with time. Longer labeling (12 h) showed evidence of rRNA, visible as two small peaks at approximately 41 and 44 s, which is generally synthesized at a slower rate than mRNA [34]. Pre-existing RNA profiles (Figure 2b) looked similar to typical *K. brevis* total RNA profiles with prominent ribosomal peaks at approximately 41 and 44 s and numerous smaller RNAs visible. Both newly synthesized and pre-existing fractions of *K. brevis* RNA were of high quality and amenable to downstream applications such as microarray and qPCR.

### Global analysis of *K. brevis* mRNA reveals unusually stable half-lives

To establish the global distribution of RNA half-lives in *K. brevis*, we utilized the method of Dölken et al. [22] as modified by Friedel and Dölken [28]. Six liters of *K. brevis* were labeled with 4tU for 2 h and total RNA was biotinylated and separated into 3 fractions: total RNA, pre-existing RNA, and newly synthesized RNA. Each fraction from each replicate was Cy3 labeled and hybridized to an Agilent custom microarray for *K. brevis* containing 10,263 unique 60-mer gene probes. This data has been deposited in the Gene Expression Omnibus (GEO) as accession # GSE46174. An array p-value of p≤0.0001 was required on all 18 arrays for inclusion of a probe in half-life calculations, resulting in 7102 probes used for the analysis in the freely available software HALO [31]. Unlike typical gene expression microarray analyses, the input RNA from total, pre-existing, and newly synthesized RNA pools is not equivalent, and therefore direct comparisons of probe intensity cannot be made across arrays. However, because the newly transcribed and pre-existing RNA together make up total RNA, a negative correlation should be observed between their ratios to total RNA; thus, HALO utilizes a linear regression of these ratios to normalize array data. Following regression normalization in HALO, data were further filtered such that outliers were removed from further analyses, resulting in a final data set of 7086 features. Regression plots of the raw and normalized ratios are provided in Figure S1. Using the normalized values from linear regression, RNA half-lives can then be calculated using any one of three ratios: newly synthesized/total RNA, pre-existing/total RNA, or newly synthesized/pre-existing RNA. In this study we chose to use the ratio of newly synthesized/pre-existing RNA ratio because it combines the overall performance of the newly synthesized/total RNA with the higher precision obtained from the pre-existing/total RNA ratio for messages with short half-lives [28], [31].

**Figure 3:**
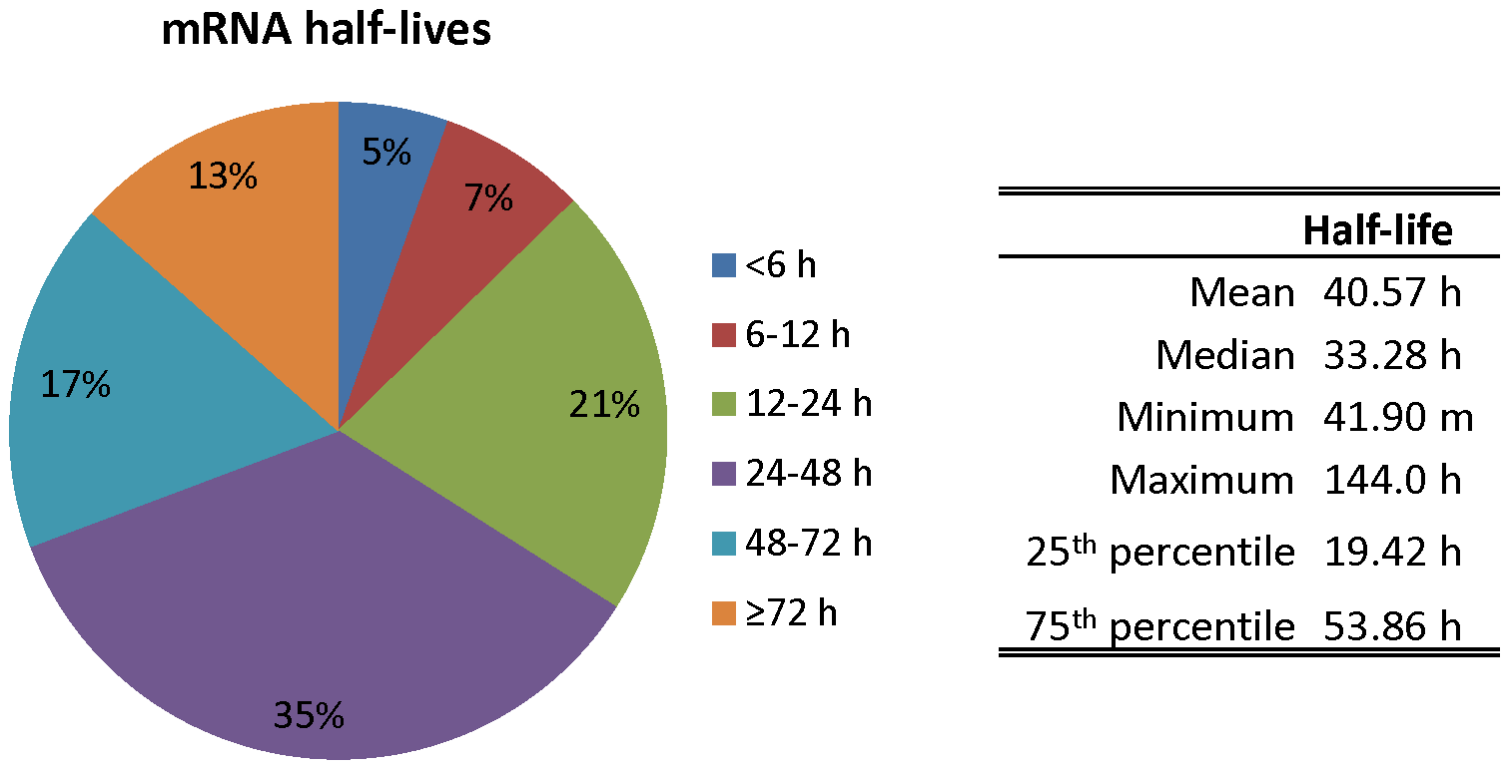
*Karenia brevis* mRNA half-lives. Following 2 h incubation with 0.2 mM 4-thiouracil, total RNA was biotinylated and bead purified. Total, pre-existing, and newly synthesized RNA fractions were hybridized to a custom oligonucleotide microarray and mRNA half-lives were calculated for 7086 features using HALO. Following normalization by linear regression, newly synthesized RNA to pre-existing RNA ratios were used to calculate half-lives.

Overall, extremely stable messages were observed, with a mean of 40.6 h and median of 33.3 h for the 7086 features (Figure 3; Table S2). The minimum half-life calculated was 41.9 m, while 1.4% of features reported a half-life of at least 6 days (Figure 3, Table S2). Over 13% of the features had a half-life of at least 3 days, whereas only 5.4% of features had half-lives of less than 6 h (Figure 3). Half-lives were also calculated using pre-existing/total RNA ratios and newly synthesized/total RNA ratios with similar results (Figure S2). A highly significant correlation was observed between data sets calculated using newly synthesized/pre-existing RNA and newly synthesized/total RNA ratios (Spearman's Rho  = 0.9811, p<0.0001, n = 7086). The correlation between half-life calculations based on newly synthesized/pre-existing RNA and pre-existing/total RNA was much lower (Spearman's Rho = 0.4811, p<0.0001, n = 7086). This is consistent with the results of Friedel and Dölken [28], who found that that half-lives calculated from pre-existing to total RNA ratios are precise for short-lived messages, but unreliable for stable messages.

The median half-life of *K. brevis* transcripts calculated in this study exceeded that of other organisms reported to date. Global analyses of transcript stability report median mRNA half-lives of 2.4 m in the cyanobacteria *Prochlorococcus* [43] and 5 m in *E. coli* [44]. Among eukaryotes, the median mRNA half-life in yeast was found to be 21 m [45], *Arabidopsis* 3.8 h [46]; mouse 7.1 h [27], and human 10 h [47]. In trypanosomes, a median half-life of 13 m is found in the bloodstream form [23], but half-lives are generally much longer in the more slowly growing procyclic stage [48], [49]. Amongst most organisms studied, the median RNA half-life is roughly proportional to the length of the cell cycle [45], [46], [47]: in bacteria with a cell cycle of 20 m, the median RNA half-life is 5 m; in yeast with a cell cycle of 90 m the median RNA half-life was 21 m; in human HepG2/Bud8 cells with a cell cycle of 3000 m the median RNA half-life was 600 m. In comparison, in *K. brevis* with an average cell cycle of approximately 3 days (4320 m) [50], [51], [52], the median RNA half-life was 33.3 h (1998 m). However, in all organisms individual transcript half-lives ranged from minutes to hours or days, in many cases the lifetime of mRNA was approximately one cell generation time. Greenberg calculated that approximately 40% of mammalian mRNAs survive more than one cellular generation [53]. In the case of *K. brevis*, approximately 13% of features showed a half-life of at least 3 days, or at least one generation time. It has been noted in trypanosomes that messages present in high copy number tend to be more stable, while rarer messages have short half-lives [23]. As the *K. brevis* microarray employed in these studies was constructed using 10,263 unique contigs obtained from Sanger sequencing, it is possible that the transcriptome features arrayed are biased towards the more highly expressed transcripts. With a genome size of 1×10^11^ bp, *K. brevis* is predicted to possess approximately 75,000 expressed genes [2]. Indeed, preliminary analyses of a deep transcriptome sequencing of *K. brevis* using the Illumina sequencing platform resulted in over 75,000 unique contigs (Van Dolah and Morey, unpublished). Thus, the absence of extremely short-lived mRNAs in this study may not preclude their occurrence in *K. brevis*; rather those sequences may not be represented on the microarray employed. A similar analysis using RNA-seq may reveal a missing population of shorter-lived messages. It is also plausible that the unusually long half-lives reported here reflect another characteristic of dinoflagellate physiology: circadian regulation. Many processes in dinoflagellates are under circadian control, including bioluminescence [10], [11], [12], photosynthesis [13], and the cell cycle [15], [16]. In *Lingulodinium polyedrum*, translation also appears to occur almost exclusively during the subjective night [54]. Thus, it is conceivable that messages are stabilized throughout the day pending their translation at night. Since the current study was carried out during the light phase of the diel cycle, and the method of half-life calculation assumes steady state, a nighttime turnover of mRNA would not be detected and cannot be discounted.

**Figure 4:**
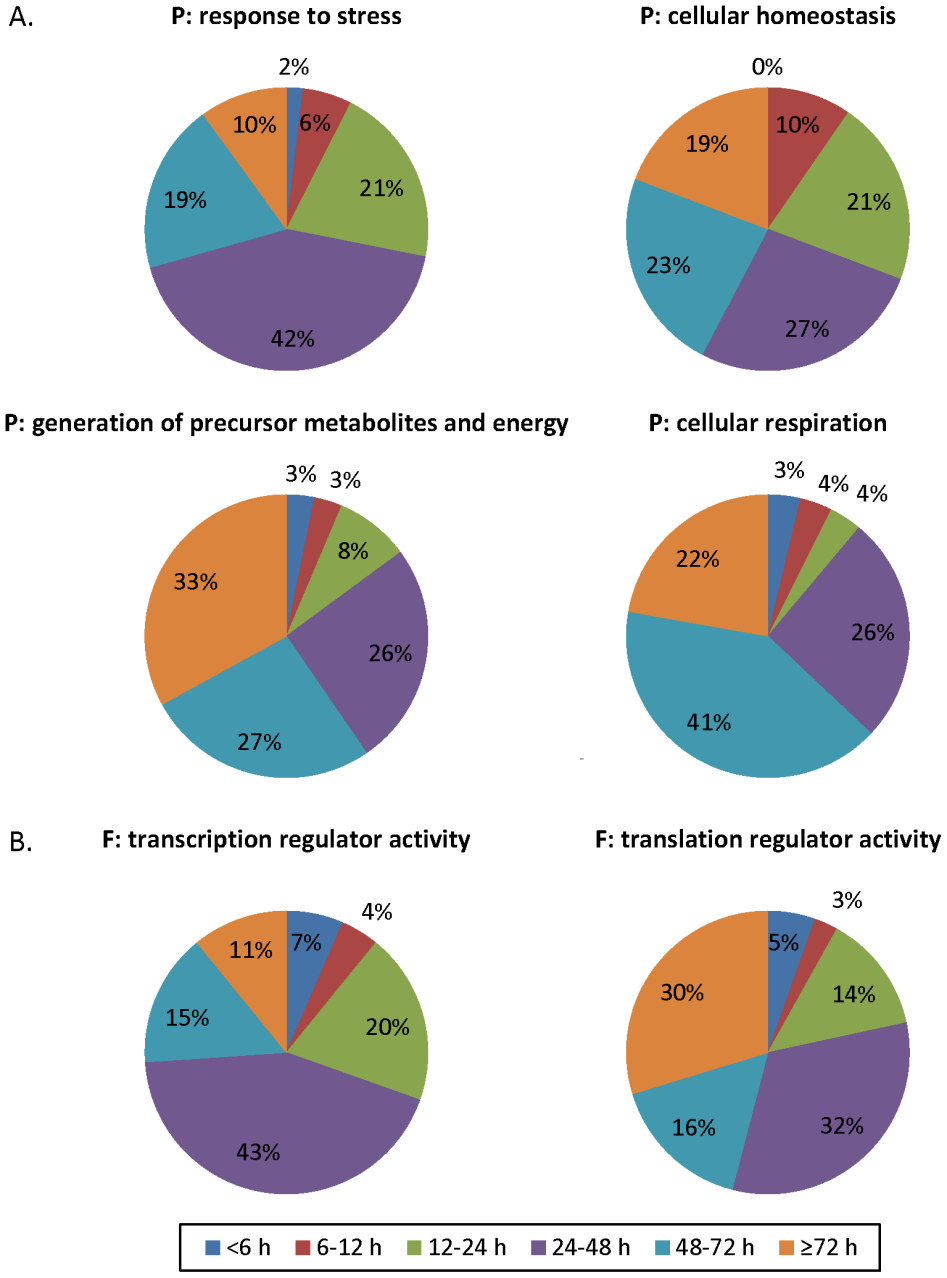
mRNA half-life distributions for selected GO terms. Gene ontology terms were assigned using Blast2GO Yeast GO slim. mRNA half-lives were binned on time and distributions are shown for selected GO terms. **A.** Biological Process GO terms. GO:0006950, response to stress; GO:0019725, cellular homeostasis; GO:0006091, generation of precursor metabolites and energy; GO:0045333, cellular respiration. **B.** Molecular Function GO terms. GO:0030528, transcription regulator activity; GO:0045182, translation regulator activity.

An emerging pattern of mRNA half-lives in organisms studied at the global level reveals that genes involved in regulatory/response functions (e.g. regulation of transcription and signal transduction) and stress responses are among the least stable messages, enabling the cell to rapidly alter their abundance through alternative synthesis and degradation. In contrast, genes involved in housekeeping functions (e.g. energy metabolism, cellular respiration, structure) are overall among the most stable messages, alleviating the need to continually synthesize new messages for constitutive processes [24], [27], [44], [45], [46]. To assess if these conserved principles hold true for *K. brevis*, we utilized Fisher's Exact tests for enrichment in Blast2GO on the binned *K. brevis* mRNA half-life data. Significant enrichment was only observed among mRNAs with a half-life of at least 3 days (Table 1). Indeed, we observed significant over-enrichment of several GO functions and processes including energetic processes, metabolism, and transport. Significant enrichment was not observed among transcripts with shorter half-lives in *K. brevis*. However, when the distribution of mRNA half-lives was compared qualitatively between GO terms, it was apparent that the *K. brevis* half-lives concurred with observations from other species. For example, when querying biological process Yeast GO slim terms, only 10% of features with an annotation of response to stress had a half-life of at least 3 days (Figure 4a). In contrast, among other biological process Yeast GO slim terms of cellular homeostasis, generation of precursor metabolites and energy and cellular respiration, 19–33% of features had half-lives of greater than 3 days. Likewise the distribution of *K. brevis* mRNA half-lives involved in the molecular function Yeast GO slim terms of transcription regulator activity and translation regulator activity agreed with the observations in mammalian cells, wherein mRNAs encoding for transcription regulation were less stable and half-lives for transcripts involved in translation regulation were somewhat longer [24]. In the current study 11% of features involved in transcription regulator activity had at least a 3 day half-life, compared to 30% of features involved in translation regulator activity (Figure 4b).

**Table 1:** Significantly over-enriched gene ontology (GO) categories, assigned using the standard GO vocabulary, among features with calculated half-lives of at least 3 days.

GO ID	GO Term	Category^a^	FDR^b^
GO:0045263	proton-transporting ATP synthase complex, coupling factor F(o)	C	0.0253
GO:0033177	proton-transporting two-sector ATPase complex, proton-transporting domain	C	0.0253
GO:0045259	proton-transporting ATP synthase complex	C	0.0253
GO:0046933	hydrogen ion transporting ATP synthase activity, rotational mechanism	F	0.0253
GO:0022804	active transmembrane transporter activity	F	0.0253
GO:0046961	proton-transporting ATPase activity, rotational mechanism	F	0.0253
GO:0055085	transmembrane transport	P	0.0253
GO:0006091	generation of precursor metabolites and energy	P	0.0272
GO:0016469	proton-transporting two-sector ATPase complex	C	0.0332
GO:0005743	mitochondrial inner membrane	C	0.0332
GO:0019866	organelle inner membrane	C	0.0332
GO:0019829	cation-transporting ATPase activity	F	0.0332
GO:0015405	P-P-bond-hydrolysis-driven transmembrane transporter activity	F	0.0332
GO:0015399	primary active transmembrane transporter activity	F	0.0332
GO:0046034	ATP metabolic process	P	0.0332
GO:0010608	posttranscriptional regulation of gene expression	P	0.0332
GO:0006754	ATP biosynthetic process	P	0.0332
GO:0015986	ATP synthesis coupled proton transport	P	0.0332
GO:0015985	energy coupled proton transport, down electrochemical gradient	P	0.0332
GO:0034220	ion transmembrane transport	P	0.0332
GO:0006818	hydrogen transport	P	0.0353
GO:0015992	proton transport	P	0.0353
GO:0031966	mitochondrial membrane	C	0.0358
GO:0031967	organelle envelope	C	0.0402
GO:0015078	hydrogen ion transmembrane transporter activity	F	0.0402
GO:0009206	purine ribonucleoside triphosphate biosynthetic process	P	0.0402
GO:0009145	purine nucleoside triphosphate biosynthetic process	P	0.0402
GO:0051246	regulation of protein metabolic process	P	0.0462
GO:0008289	lipid binding	F	0.0463
GO:0009201	ribonucleoside triphosphate biosynthetic process	P	0.0483
GO:0009142	nucleoside triphosphate biosynthetic process	P	0.0483
GO:0005740	mitochondrial envelope	C	0.0500

a C: Cellular Component, F: Molecular Function, P: Biological Process.

b Reported FDR values are from a Fisher's Exact Test in Blast2GO.

**Figure 5:**
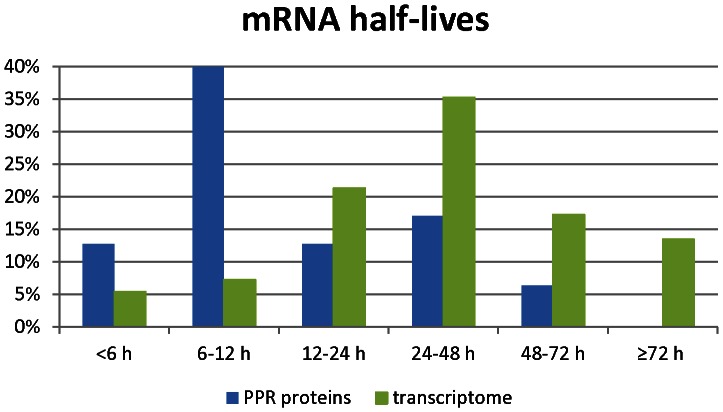
mRNA half-live distributions for pentatricopeptide repeat (PPR) protein transcripts. mRNA half-lives were calculated for 47 of the 93 features annotated as PPRs on the array. mRNA half-lives were binned on time and distributions are shown for these PPR transcripts. The distribution of 7086 features of the *K. brevis* transcriptome are re-plotted from figure 3 for ease of comparison.

Among the genes involved in RNA processing in *K. brevis* is a highly represented family of pentatricopeptide repeat (PPR) containing proteins. PPR proteins are sequence-specific RNA binding proteins involved in post- transcriptional processing of organellar RNAs, including editing, splicing, translation, and stability [55], [56], [57]. These transcripts are of particular interest because they are among the earliest transcripts to change in abundance following nutrient addition to nutrient starved cultures [18], consistent with their function in regulating organellar RNA necessary for renewed photosynthetic activity. We queried the mRNA half-lives calculated for 47 of the 93 features annotated as PPR proteins on the microarray. The minimum half-life was 97 m and the maximum 70 h, with a median half-life of 9.1 h. The distribution is greatly skewed to shorter half-lives (Figure 5), with 64% of PPRs having half-lives <12 h, in comparison to only 12% of features in the complete data set. Thus, as observed in *Arabidopsis* [46], PPRs in *K. brevis* have much shorter half-lives than the transcriptome as a whole. These short half-lives for PPRs are likely indicative of their essential roles in regulatory functions in chloroplasts and mitochondria [46], [55]. As short-lived transcripts, they are primed for immediate response to perturbations as observed our previous microarray studies following N- and P-addition [18].

**Figure 6:**
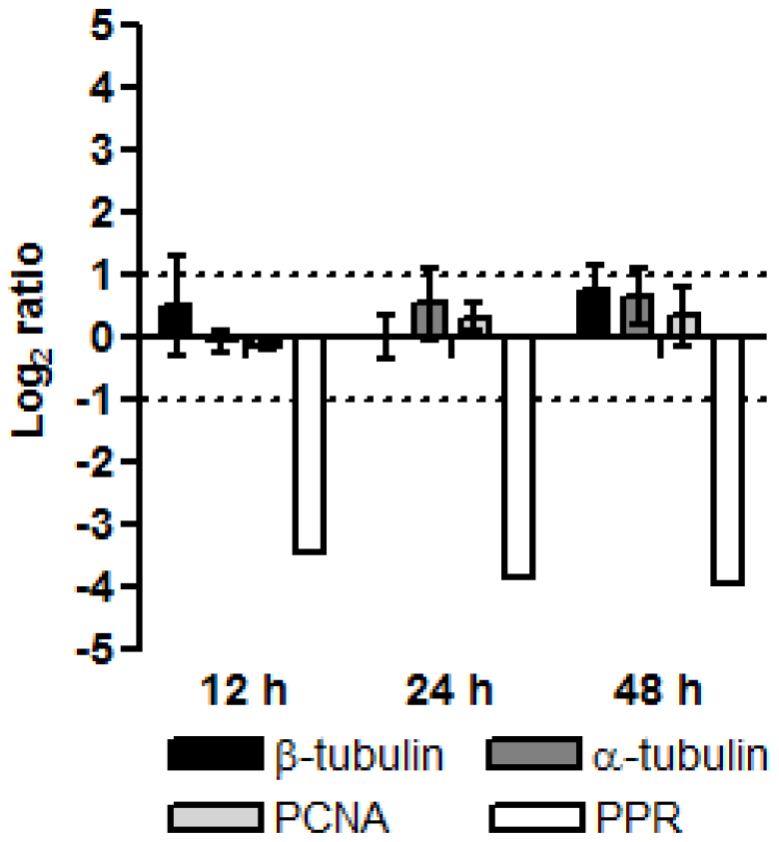
Stability of selected mRNAs following transcription inhibition assessed by qPCR. Triplicate cultures were treated with 2 µg·mL^−1^ actinomycin D for 12, 24, or 48 h and the transcript abundance determined relative to DMSO carrier controls at each time point. Means ±SD (n = 3) are plotted. The PPR transcript was undetected in two of three replicates at 24 and 48 h following 40 PCR cycles, therefore SDs were not calculated. Dashed lines represent a 2-fold change.

To independently assess half-lives of selected RNAs, we used the traditional approach of blocking transcription with actinomycin D and following transcript levels over a time course of 12, 24 and 48 h by qPCR. We selected two cytoskeletal genes, α-tubulin and β-tubulin, one cell cycle gene, PCNA, previously shown to be regulated in *K. brevis* by post-transcriptional mechanisms [15], and a PPR protein (Figure 6). The PPR protein transcript dramatically declined in abundance by 12 h, relative to its abundance in time matched controls, with almost a complete loss of transcript by 24 h (undetected after 40 qPCR cycles in 2 of 3 replicates). The half-life for this PPR protein (Contig 3257) was calculated to be just under 7 h in the microarray study, indicating a relatively short half-life compared to the transcriptome as a whole. In contrast, none of the other transcripts showed a decrease in abundance even after 48 h, suggesting that the tubulin and PCNA transcripts are highly stable. Three α-tubulin probes on the array reported a mean half-life of 38.4 h, four probes for β-tubulin reported a mean half-life of 64.1 h, while a PCNA probe reported a half-life of 27.4 h using the biosynthetic labeling approach. Although not in precise agreement, both methods identified long half-lives in the cytoskeletal genes and PCNA. Furthermore, the qPCR results lend support to the prediction that PPR repeat transcripts have short half-lives, consistent with their regulatory roles. We are aware of only one previous study that addressed message stability in dinoflagellates, in which the half-lives of circadian GAPDH, luciferin binding protein, and luciferase in *Lingulodinium polyedrum* varied somewhat depending on the time of day at which the transcription inhibitor thiolutin was added, but all were on the order of several hours as assessed by northern blot analysis [58].

### Increased de novo transcription does not mediate short term changes in transcript abundance

The presence of the SL on all nuclear encoded gene transcripts examined in dinoflagellates, along with the observation of post-transcriptional regulation of a number of processes studied, has led to the hypothesis that gene regulation in dinoflagellates is dependent largely on post-transcriptional processes [9]. Consistent with this, a microarray study of the *K. brevis* transcriptome's response to nutrient addition found only 4.4% of array features significantly changing over a 48 h time course following nitrate addition to N-depleted *K. brevis* cultures [18]. However, minimal changes were observed early in the time course with only 0.38% of array features significantly changed in abundance 4 h post N-addition. Intriguingly, among these early changers nearly 50% of the annotated features were PPR proteins. This raised the question as to whether PPR proteins are a class of genes that are under transcriptional regulation. Typical microarray experiments cannot resolve if changes in message abundance are due to *de novo* transcription and or to changes mRNA stability. Therefore, in the current study we examined *de novo* transcription by 4tU incorporation into newly synthesized RNA over two 1 h periods (0–1 h and 3–4 h) following N addition in comparison with that in the N-depleted cultures This data has been deposited as GEO Accession # GSE46175.

During the first hour post N-addition only 0.2% of array features were significantly different in the newly transcribed RNA pool when compared to the N-depleted cells similarly labeled for 1 h (≥1.7 fold change and p-value ≤0.0001) (Table 2). However, 15 of the 21 significantly changing features were less abundant than in the N-depleted cultures, suggesting their rate of transcription decreased. The number of significantly different genes increased to 0.34% of array features during the fourth hour post N-addition (Table 3), a percentage very similar to that reported in the previous whole transcriptome study [18]. By this time 22 of the 36 significant array features had increased in abundance. Overall, the small number of differentially expressed array features among newly transcribed RNA queried in this study suggests that there was limited transcriptional activation involved in the early response to N-addition. No PPR protein transcripts showed significant changes in abundance in the newly synthesized RNA pool relative to the N-depleted controls at either time point. Thus it appears that changes in transcription rate did not contribute to the previously observed increase PPR protein transcript abundance. This and the presence of the SL sequence on these PPR repeat protein messages [18] supports the hypothesis that their change in expression was achieved through differential message stability.

**Table 2:** Array features with significantly different abundance during the first hour post-nitrate addition, relative to N-depleted cultures.

Contig	Description	BLASTx e-value	fold change	p-value
10845	hCG1994859	1.80E−01	33.97	3.70E−09
5509	PREDICTED: hypothetical protein LOC100401915	8.98E−05	21.55	2.24E−05
1269	chloroplast light harvesting protein isoform 12	8.81E−13	15.88	1.57E−06
9771	membrane bound adenylate/guanylate cyclase with PAS domain	1.02E−07	11.40	2.07E−06
**8807**	COG2307: Uncharacterized protein	8.50E−01	2.48	1.29E−05
**9812**	glutamine synthetase	7.30E−02	2.05	9.89E−05
8060	PREDICTED: hypothetical protein	2.90E+00	−1.70	2.72E−25
6696	regulatory protein, putative	4.80E+00	−1.72	3.99E−08
**6529**	UOS5/S1 protein, putative	2.26E−08	−1.83	8.64E−05
7967	COG3119: Arylsulfatase A	1.40E+00	−1.85	2.76E−05
10741	CPA1 family transporter: sodium ion/proton	7.80E−01	−1.85	2.68E−05
3375	hypothetical protein Kpol_376p14	8.00E−03	−2.17	1.28E−06
6519	h-caldesmon	4.00E−05	−2.84	1.91E−06
11662	serine threonine-specific protein phosphatase	1.00E−24	−7.78	3.62E−05
10272	60S acidic ribosomal protein P0	3.00E−54	−9.18	8.87E−06
5289	von willebrand factor type a domain protein	1.84E−23	−11.03	9.50E−06
**2363**	eukaryotic translation initiation factor 5A	2.55E−20	−12.20	6.72E−06
9695	putative sugar transport membrane protein	1.80E+00	−12.78	4.16E−05
10482	hypothetical protein OsI_030791	1.80E−01	−16.20	7.56E−05
**7355**	AChain A, Transposase Inhibitor	1.20E−168	−16.21	2.35E−07
2545	conserved hypothetical protein	1.40E+00	−27.35	7.21E−09

Contigs in bold are also significantly different during the fourth hour post-nitrate addition.

**Table 3:** Array features with significantly different abundance during the fourth hour post-nitrate addition, relative to N-depleted cultures.

Contig	Description	BLASTx e-value	fold change	p-value
8061	hypothetical protein BoklE_05717	8.10E−01	18.96	2.14E−06
**9812**	glutamine synthetase	7.30E−02	4.26	7.49E−08
**8807**	COG2307: Uncharacterized protein conserved	8.50E−01	2.98	1.57E−05
10877	Peroxisomal catalase	2.50E+00	2.81	8.45E−06
6074	Extracellular ligand-binding receptor	5.70E−01	2.51	2.20E−06
5893	Tetratricopeptide TPR_2 repeat protein	2.00E−06	2.45	2.32E−05
5722	putative arsenate reductase	2.20E+00	2.41	1.24E−05
797	neuropilin	2.30E+00	2.37	3.12E−05
3750	merozoite surface protein 3 (MSP3), putative	8.60E−02	2.37	1.48E−05
10903	type I polyketide synthase-like protein KB4825	3.31E−121	2.17	4.02E−05
2927	Malonyl-CoA:ACP transacylase (ISS)	8.00E−30	2.13	1.30E−06
118	Malonyl-CoA:ACP transacylase (ISS)	2.00E−51	2.07	1.33E−05
8273	hypothetical protein CaO19.4072	1.70E−02	1.97	1.27E−10
8698	type I polyketide synthase-like protein KB6842	4.79E−177	1.95	1.55E−06
851	hypothetical protein SSO2408	2.60E−01	1.92	5.58E−06
1739	heat domain-containing protein	1.69E−14	1.90	2.18E−05
964	PREDICTED: similar to 4633402D15Rik protein	1.90E+00	1.87	1.06E−08
9237	type I polyketide synthase-like protein KB5361	9.56E−111	1.80	9.96E−05
5784	Cupin 4 family protein	5.00E−09	1.78	1.49E−12
9799	protein arginine N-methyltransferase-like protein	4.00E−12	1.77	2.44E−08
3629	PREDICTED: similar to hypothetical gene	1.10E−01	1.74	5.36E−05
9473	chaperone protein	3.40E−01	1.71	5.79E−05
**6529**	3CCCH domain containing protein	4.00E−06	−1.77	4.72E−08
10990	alpha-1,3-mannosyl-glycoprotein 4-beta-N-acetylglucosaminyltransferase C	6.38E−32	−1.93	5.66E−06
10449	Putative membrane protein	1.60E−01	−2.16	1.97E−05
11834	*No hits found*	-	−2.27	1.55E−05
8815	DNA-binding protein	1.30E−01	−2.86	2.44E−07
4388	alanine aminotransferase	8.25E−60	−2.94	9.76E−05
7379	putative formate dehydrogenase	3.90E−02	−3.56	5.55E−05
5143	Kynurenine 3-monooxygenase and related flavoprotein monooxygenases (ISS)	6.32E−10	−3.75	3.18E−05
6738	ATP-binding component of molybdate transporter	3.00E−01	−6.94	8.49E−13
4218	glycoside hydrolase family 3 domain protein	4.01E−26	−13.28	8.57E−09
**7355**	AChain A, Transposase Inhibitor	1.20E−168	−14.15	4.94E−05
**2363**	eukaryotic translation initiation factor 5A	2.55E−20	−18.93	1.74E−07
4062	proteophosphoglycan ppg4	2.00E−05	−19.21	1.04E−07
6079	calcium-dependent protein kinase 33	4.17E−07	−24.25	4.79E−06

Contigs in bold are also significantly changing during the first hour post-nitrate addition.

## Conclusions

Insight into mRNA stability is important to understanding gene regulation, particularly in poorly characterized and divergent eukaryotes. Here we demonstrate that the pyrimidine salvage pathway of the dinoflagellate, *K. brevis*, is capable of incorporating 4-thiouracil into newly transcribed RNA, making it amenable to studying RNA stability using biosynthetic labeling approaches. By microarray analysis, *K. brevis* appears to possess highly stable messages relative to other eukaryotic models. Further analyses using deeper transcriptome surveys via RNA-seq may refine this estimation of median half-life. In addition, the potential contribution of circadian accumulation and turnover of messages must be addressed. Nonetheless, in keeping with conserved properties in other eukaryotes, the dinoflagellate's most stable messages are largely involved in core processes such as energy generation and transport, whereas regulatory processes, including transcription regulation and stress response, are more highly represented among the shorter-lived transcripts. In particular, PPR repeat proteins involved in organellar RNA regulation are highly represented among the short-lived mRNAs, consistent with their rapid increase in abundance following nutrient stimulation. However, their apparent lack of transcriptional activation, along with the presence of the SL on PPR protein messages, lends support to the growing consensus of post-transcriptional control of dinoflagellate gene expression.

### NOAA Disclaimer

This publication does not constitute an endorsement of any commercial product or intend to be an opinion beyond scientific or other results obtained by the National Oceanic and Atmospheric Administration (NOAA). No reference shall be made to NOAA, or this publication furnished by NOAA, to any advertising or sales promotion which would indicate or imply that NOAA recommends or endorses any proprietary product mentioned herein, or which has as its purpose an interest to cause the advertised product to be used or purchased because of this publication.

## Supporting Information

Figure S1 **Regression plots used for normalization in HALO.** Following 2 h incubation with 0.2 mM 4-thiouracil, total RNA was biotinylated and bead purified. Total, pre-existing, and newly synthesized RNA fractions were hybridized to a custom oligonucleotide microarray. Linear regressions of the ratios of newly synthesized RNA to total RNA and pre-existing RNA to total RNA are conducted in HALO prior to half-life calculations. The regression of the raw uncorrected data (**A**) and regression-normalized data (**B**) used for half-life calculations are shown.
(TIF)

Figure S2 ***Karenia brevis*** **mRNA half-lives calculated from additional ratios.** Following 2 h incubation with 0.2 mM 4-thiouracil, total RNA was biotinylated and bead purified. Total, pre-existing, and newly synthesized RNA fractions were hybridized to a custom oligonucleotide microarray and mRNA half-lives were calculated for 7086 features using HALO. Following normalization by linear regression, newly synthesized RNA to total RNA (**A**) or pre-existing to total RNA (**B**) ratios were used to calculate half-lives.
(TIF)

Table S1 **Primers used for quantitative real-time PCR.**
(XLSX)

Table S2 **Half-lives for 7086 features passing quality filters based on newly synthesized RNA to pre-existing RNA ratios.**
(XLSX)
