# Supplementary figures and images for: Global Analysis of mRNA Half-Lives and de novo Transcription in a Dinoflagellate, Karenia brevis
Source: PLoS One. 2013 Jun 11;8(6):e66347. doi: 10.1371/journal.pone.0066347 (PMC3679056; doi:10.1371/journal.pone.0066347)

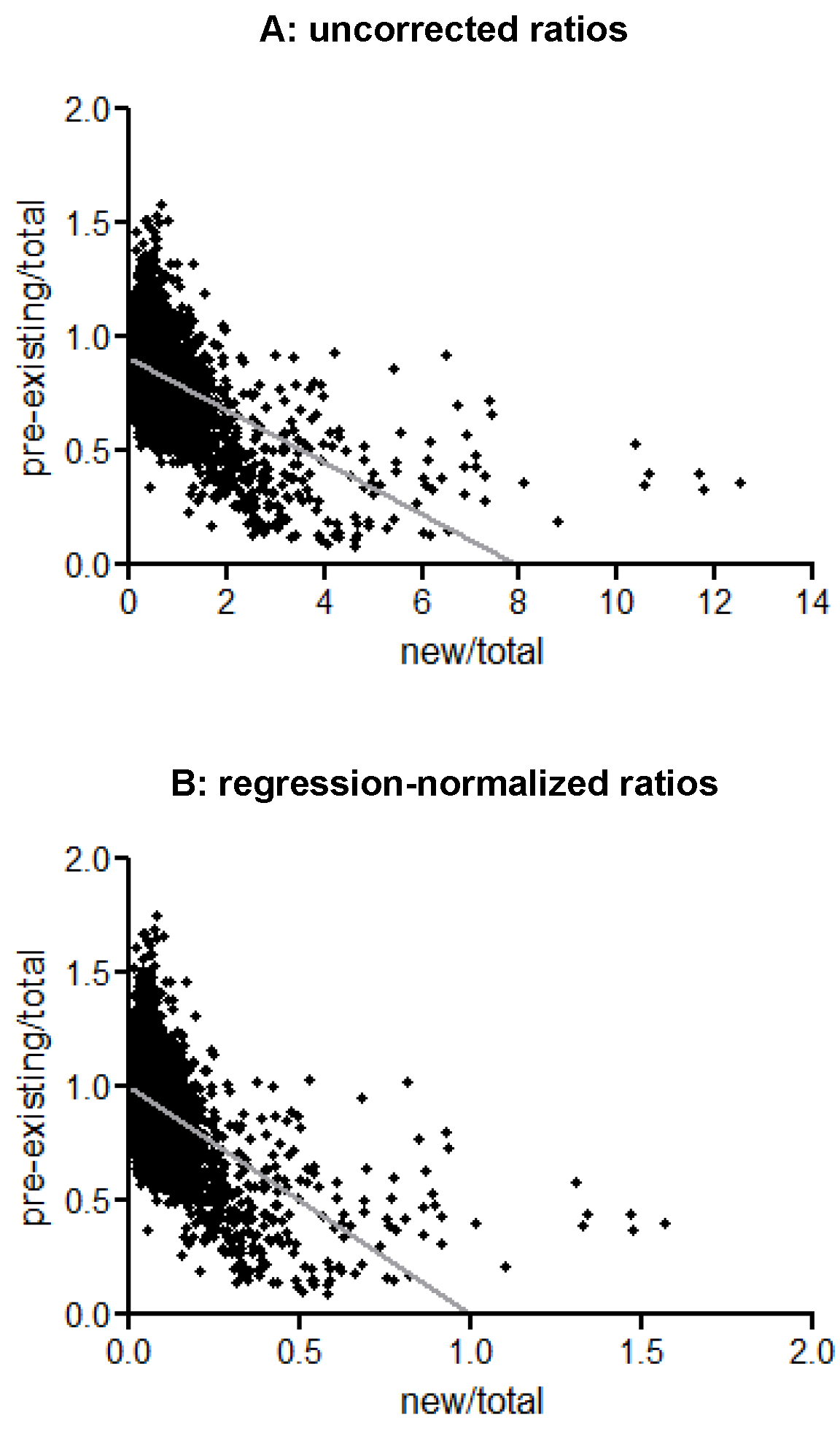

Supplement: Figure S1 — Regression plots used for normalization in HALO. Following 2 h incubation with 0.2 mM 4-thiouracil, total RNA was biotinylated and bead purified. Total, pre-existing, and newly synthesized RNA fractions were hybridized to a custom oligonucleotide microarray. Linear regressions of the ratios of newly synthesized RNA to total RNA and pre-existing RNA to total RNA are conducted in HALO prior to half-life calculations. The regression of the raw uncorrected data (A) and regression-normalized data (B) used for half-life calculations are shown. (TIF) [file pone.0066347.s001.tif]

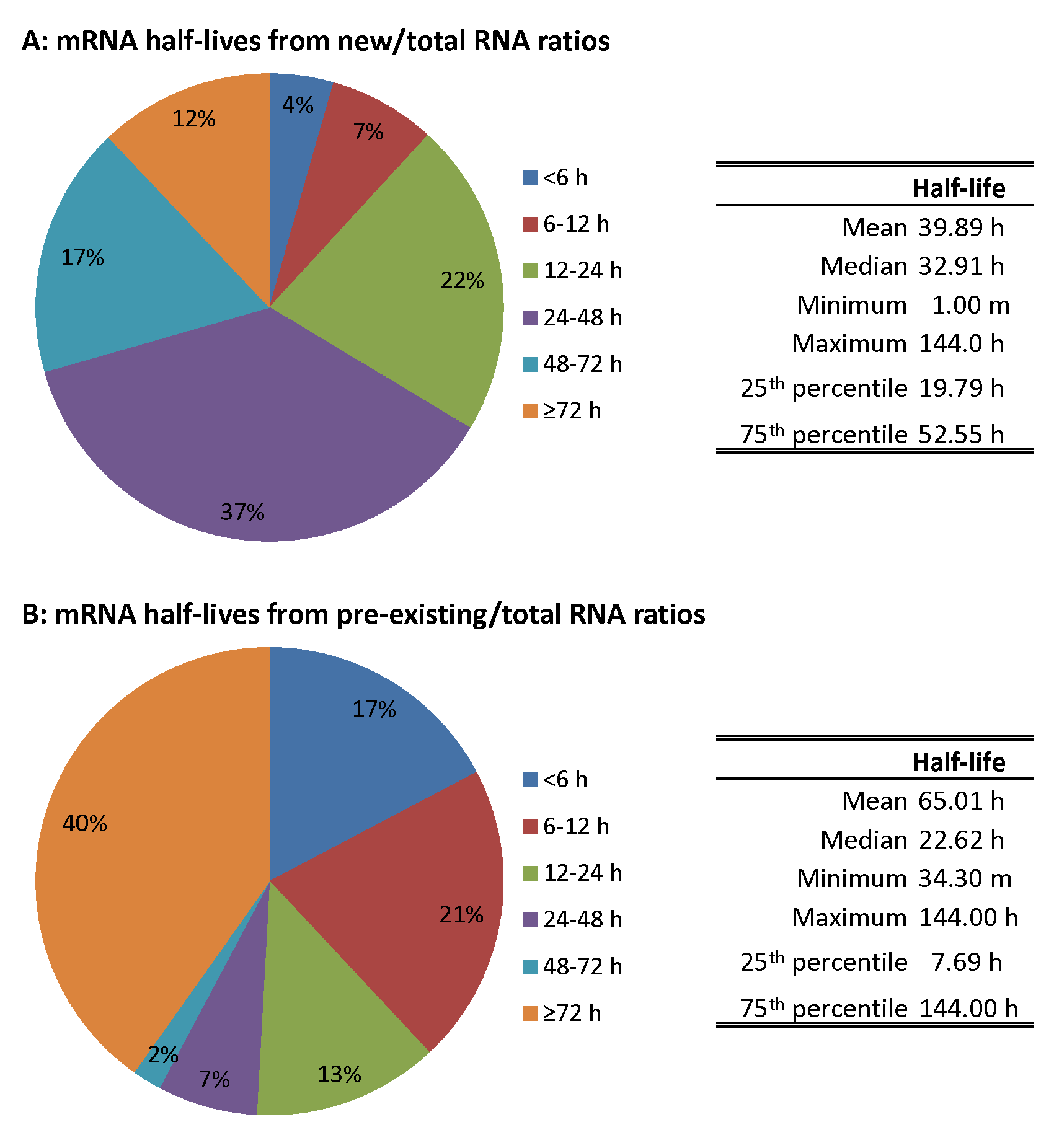

Supplement: Figure S2 — Karenia brevis mRNA half-lives calculated from additional ratios. Following 2 h incubation with 0.2 mM 4-thiouracil, total RNA was biotinylated and bead purified. Total, pre-existing, and newly synthesized RNA fractions were hybridized to a custom oligonucleotide microarray and mRNA half-lives were calculated for 7086 features using HALO. Following normalization by linear regression, newly synthesized RNA to total RNA (A) or pre-existing to total RNA (B) ratios were used to calculate half-lives. (TIF) [file pone.0066347.s002.tif]
